# Reviewer Acknowledgement 2012

**DOI:** 10.1186/1471-2164-14-231

**Published:** 2013-05-02

**Authors:** Catherine M Rice

**Affiliations:** 1 BioMed Central, 236 Gray's Inn Road, London, WC1X 8HB, UK

## Abstract

**Contributing reviewers:**

The editors of *BMC Genomics* would like to thank all our reviewers who have contributed to the journal in volume 13 (2012).

## 

Reidunn Aalen

Norway

Behnam Abasht

USA

Michael Abberton

UK

Jessica Abbott

Sweden

Hesham Abdeen

Egypt

Mona Abirached-Darmency

France

Mourad Aboul-Soud

Egypt

Tilmann Achsel

Belgium

Charles Addo-Quaye

USA

Saulo Aflitos

Netherlands

Samuel E Aggrey

USA

Xabier Agirre

Spain

Nisar Ahmed

Pakistan

Niyaz Ahmed

India

Veenu Aishwarya

India

Yusuf Akhter

Germany

Eduard Akhunov

USA

Pascale Alard

USA

Arthur Alberts

USA

Ute Albrecht

USA

Lee Alexander

USA

Salem Alghamdi

Saudi Arabia

Maisa Ali

Kuwait

Can Alkan

USA

Elaine Allan

UK

Randy Allen

USA

Mark Alter

USA

Craig Altier

USA

Carlos Alvarez

USA

Fernando Alvarez-Valin

Uruguay

Nelson Alves Junior

Brazil

Marcio Alves-Ferreira

Brazil

Maria Antonia Amaral Turkman

Portugal

Yong-Qiang An

USA

Gynheung An

Korea, South

Vivek Anantharaman

USA

Gema Ancillo

Spain

Don Anderson

USA

Jan Andersson

Sweden

Yaroslav Andreev

Russian Federation

Daniel Andrews

Australia

Ioannis Androulakis

USA

Oñate Angel

Chile

Claudia Angelini

Italy

Matti Annala

Finland

Caroline Anselme

France

Aldwin Anterola

USA

S Yahya Anvar

Netherlands

Rudi Appels

Australia

Katsutoshi Arai

Japan

Kazuharu Arakawa

Japan

Maria José Aranzana

Spain

Marcos J. Arauzo-Bravo

Germany

Filippos Aravanopoulos

Greece

Tzahi Arazi

Israel

Peter Arensburger

USA

Juan Lucas Argueso

USA

Stilianos Arhondakis

Greece

Masanori Arita

Japan

John Armour

UK

Ian Armstead

UK

Catherine Arnold

UK

David Arnosti

USA

Randolph Arroo

UK

Pere Arus

Spain

Mikko Arvas

Finland

David Askew

USA

Yan Asmann

USA

Juan F. Asturiano

Spain

Nadia Aubin-Horth

Canada

Benjamin Audit

France

Paul Auer

USA

Raymond Auerbach

USA

Ingo Autenrieth

Germany

Karen Avraham

Israel

Michael Axtell

USA

Koichiro Aya

Japan

Belay Ayele

Canada

Julien Ayroles

USA

Melanie Babcock

USA

Igor Babiak

Norway

Jaume Bacardit

UK

Doris Bachtrog

USA

Tsvetan Bachvaroff

USA

Jonathan Badger

USA

Timothy Bailey

Australia

Matthew Bainbridge

USA

Harsh Bais

Sudan

Michael Baitaluk

USA

Lauren Bakaletz

USA

Guus Bakkeren

Canada

Angel Baldan

USA

Laura Baldo

Switzerland

William Baldwin

USA

Eldon Ball

Australia

Richard Baltz

USA

Piotr Jan Balwierz

Switzerland

Mark Banfield

UK

Manju Bansal

India

Abdelali Barakat

USA

Yoseph Barash

USA

Daniel Barbash

USA

Nuno L Barbosa-Morais

Canada

Jason Barbour

USA

J Christopher Bare

USA

William Barendse

Australia

Luca Bargelloni

Italy

Linda Barlow

USA

Joanne Barnes Weidhaas

USA

Ross Barnett

UK

Francisco Barona-Gomez

Mexico

Younas Barozai

Pakistan

Fx Barre

France

Carlos Barreiro

Spain

Roberto Antonio Barrero

Australia

Craig Barrett

USA

Daniel Barshis

USA

Artem Barski

USA

Andreas Barth

USA

Eva Bartova

Czech Republic

Patrick Bastien

France

Jacqueline Batley

Australia

Cristina Battaglia

Italy

Peggy Baudouin-Cornu

France

Florian F Bauer

South Africa

Stefan Bauersachs

Germany

Iliana Baums

USA

Line Bay

Australia

Ken Bayles

USA

Baltazar Becerril

Mexico

David Beck

USA

Thomas Becker

Australia

David Begun

USA

Jerzy Behnke

UK

Susanta Behura

USA

Tim Beissbarth

Germany

Stefan Bekiranov

USA

Dorte Bekkevold

Denmark

Faith Belanger

USA

Stephen Bell

UK

Matthew Bellgard

Australia

Arianna Bellucci

Italy

Kathy Belov

Australia

Alexa Bely

USA

Andrew Bendall

Canada

Celso Benedetti

Brazil

Vladimir Benes

Germany

Cesar Benito

Spain

Yuval Benjamini

USA

George Bennett

USA

Sasisekhar Bennuru

USA

Panayiotis (Takis) Benos

USA

Stephen Bentley

UK

Andrew Bergen

USA

Sara Bergonzi

Germany

Renaud Berlemont

USA

Stewart Berlocher

USA

Rolf Bernander

Sweden

Giorgio Bernardi

Italy

Louis Bernier

Canada

Matthew Berriman

UK

Daniel Besser

Germany

Laure Beven

France

Madan Bhattacharyya

USA

Sukesh Bhaumik

USA

Christopher Bidwell

USA

Fabiana Bigi

Argentina

Hans Binder

Germany

Daniel Bird

Australia

Inanc Birol

Canada

Angela Bithell

UK

Michael Black

Australia

Jerry Blackwell

USA

Judith Blake

USA

Robert Blakesley

USA

Jose M. Blanca

Spain

Robert Blankenship

USA

Christoph Bleidorn

Germany

Matthew Blow

USA

Ralph Bock

Germany

Ulrich Bodenhofer

Austria

Mark Boekschoten

Netherlands

Martin Boer

Netherlands

Cornelius Boerkoel

USA

Jon Bohlin

Norway

Didier Boichard

France

Stephane Boissinot

USA

Leonardo Boiteux

Brazil

Zsolt Boldogkoi

Hungary

Hanna Bolibok-Bragoszewska

Poland

Aureliano Bombarely Gomez

USA

Muriel Bonnet

France

Anne Bonnieu

France

Stephanie Booth

Canada

Hester Bootsma

Netherlands

Nicole Borth

Austria

Stefania Bortoluzzi

Italy

Paul Boss

Australia

Arnaud Bottin

France

Adnane Boualem

France

Gerard Bouffard

USA

Kostantinos (Kostas) Bourtzis

Austria

Neil Bower

Australia

Jon Boyle

USA

Cameron Bracken

Australia

Chris Bradfield

USA

Stephen Bradford

Australia

William Bradshaw

USA

Katharina Braeutigam

Canada

Markus Brameier

Germany

Miguel Branco

UK

Thomas Braun

Germany

Kristen Brennan

USA

Patricia Brito

Portugal

Guy Brock

USA

Jeremy Brockes

UK

Gudrun Brockmann

Germany

Karl Broman

USA

Samantha Brooks

USA

Mikael Brosche

Finland

Susan Brown

USA

Reinhold Brückner

Germany

Edward Brummer

USA

Carmen Buchrieser

France

Gregory Buck

USA

Noel Buckley

UK

Hernan Burbano

Germany

Gertraud Burger

Canada

Gaelen Burke

USA

Fabien Burki

Canada

Andreas Burkovski

Germany

Norman Buroker

USA

Gloria Burow

USA

Ben Busby

USA

Pierre Bushel

USA

Fabian Buske

Australia

Lee Butcher

UK

Melinka Butenko

Norway

Geraldine Butler

Ireland

Ramesh Buyyarapu

USA

Nicole Bye

Australia

Leonid Bystrykh

Netherlands

Santiago Cabaleiro

Spain

Xuang Cai

China

Michael Calcutt

USA

Fanny Calenge

France

Elisabet Caler

USA

Juan Calvete

Spain

Stephen Cameron

Australia

Steven Cannon

USA

Francisco R Canton

Spain

Natasha Caplen

USA

Katia Cappelli

Italy

Claudia Carareto

Brazil

Lucia Carbone

USA

Faizal Careem

Canada

Daniela Carlisi

Italy

Piero Carninci

Japan

Laura Carrel

USA

Lorenzo Carretero-Paulet

Spain

Jason Carroll

UK

Vern Carruthers

USA

Eric Carstens

Canada

Kim Warwick Carter

Australia

Clay Carter

USA

Carla Caruso

Italy

Carlos R Carvalho

Brazil

Elena Casacuberta

Spain

Tamara Caspary

USA

Joe Cassady

USA

Isabelle Cassar-Malek

France

Ainara Castellanos-Rubio

USA

Todd Castoe

USA

Amy Caudy

Canada

Francesc Cebria

Spain

Nathalie Cella

Brazil

Rino Cella

Italy

Federica Censi

Italy

Joan Cerda

Spain

Mariateresa Cervera

Spain

Alessandro Cestaro

Italy

Pieter-Jan Ceyssens

Belgium

David Chagne

New Zealand

Jean Challacombe

USA

Agnes Chan

USA

Cheong Xin Chan

Australia

Ming-Tsair Chan

Taiwan

Amaresh Chandra

India

Hsueh-Wei Chang

Taiwan

Jeff Chang

USA

Jeffrey Chang

USA

Liwei Chang

USA

Shiaoman Chao

USA

Joe Chappell

USA

Nicolas Charlet Berguerand

France

Carole Charlier

Belgium

Emmanuelle Charpentier

Sweden

Keith Chater

UK

Mithu Chatterjee

USA

Dipankar Chatterji

India

Fayun Che

USA

Frederic Chedin

USA

Nor Chejanovsky

Israel

Jing Chen

USA

Jinming Chen

China

Jui-Fen Chen

Taiwan

Liangbiao Chen

China

Ping Chen

China

Shaoliang Chen

China

Wei Chen

Germany

Xian-Ming Chen

USA

Xiao-Ya Chen

China

Xiaoping Chen

China

Z. Chen

USA

Z. Jeffrey Chen

USA

Chun-Wen Cheng

Taiwan

Hans Cheng

USA

Lailiang Cheng

USA

Sara Cherry

USA

Ravindra Chibbar

Canada

Sridar Chittur

USA

Charles Chiu

USA

Norman Chiu

USA

Michael Cho

USA

Niki Chondrogianni

Greece

James Chong

UK

Vivek Chopra

USA

Keng-See Chow

Malaysia

Lane Christenson

USA

Pascal-Antoine Christin

USA

Jui-Hua Chu

Taiwan

Eui-Hwan Chung

USA

Ho-Ryun Chung

Germany

Salvatrice Ciccarese

Italy

M. Gonzalo Claros

Spain

Matthew Cleveland

USA

Djork Clevert

Austria

Martha Clokie

UK

Steve Clough

USA

Avner Cnaani

Israel

Gitta Coaker

USA

John Coffin

USA

Noel Oliver Ian Cogan

Australia

Ephraim Cohen

Israel

Lachlan Coin

UK

Nicholas Coleman

Australia

Michelle Colgrave

Australia

Desmond Collins

New Zealand

Andrew Collins

UK

Duncan Colquhoun

Norway

Colin Combs

USA

Ana Conesa

Spain

Ian Connerton

UK

Erin Connor

USA

Tom Conrad

Japan

Luciano Conti

Italy

Jens R. Coorssen

Australia

Giovanni Coppola

USA

Mark Corbett

Australia

Timothy Cornell

USA

Montserrat Corominas

Spain

Erico Costa

Brazil

Marcio Costa

Brazil

Valerio Costa

Italy

Maria Costantini

Italy

Laurent Couetil

USA

Patrick Covello

Canada

Mark Cowley

Australia

Robert Cramer

USA

Chris Creevey

Ireland

Martin Crespi

France

Alexandre Cristino

Australia

Quentin Cronk

Canada

Kevin Crosby

USA

Marc Cruts

Belgium

Peter Csermely

Hungary

Xinping Cui

USA

Luis Cunha

Brazil

Christina Cuomo

USA

Rosa M. Cusido

Spain

Fatima Cvrckova

Czech Republic

Mirjam Czjzek

France

Charles Czuprynski

USA

Aline Da Silva

Brazil

Andrew Dalby

UK

Colin Dale

USA

Tamas Dalmay

UK

Brian Dalrymple

Australia

Etienne G.J. Danchin

France

Thomas Dandekar

Germany

Patrick Danley

USA

Roy Danzmann

Canada

Zhang Daoyuan

China

Sabyasachi Das

USA

Sujoy Das Gupta

India

Shiladitya Dassarma

USA

Swapan Datta

India

Emily Davenport

USA

John Davey

UK

Megan Davey

UK

William Davidson

Canada

Matthew Davies

UK

Peter Davies

Canada

Ian Davis

USA

Richard Davis

USA

Thomas Davis

USA

Roberta Davoli

Italy

Simon Davy

New Zealand

Benoit Dayrat

USA

Pieter De Bleser

Belgium

Michele De Bortoli

Italy

Simone De Jong

USA

Ronnie De Jonge

Netherlands

Dirk-Jan De Koning

Sweden

Rafael R. De La Haba

Spain

Suzanne De La Monte

USA

Benildo De Los Reyes

USA

Ruud De Maagd

Netherlands

Emanuele De Paoli

Italy

Janine Deakin

Australia

Jeffrey Dean

USA

Ralph Dean

USA

Matt Deardorff

USA

Claire Deback

France

Thomas Debener

Germany

Stephane Debernard

France

Russell Dedrick

USA

Sandie Degnan

Australia

M Dejong

Netherlands

Rosa Del Campo

Spain

Massimo Delledonne

Italy

Christine Delorme

France

Regine Delourme

France

Serge Delrot

France

Constantinos Deltas

Cyprus

Erick Denamur

France

Xiuxin Deng

China

Megan Dennis

USA

Llewellyn Densmore

USA

Cynthia Derdeyn

USA

Michael Desalvo

USA

David Deshazer

USA

Christine Desmedt

Belgium

Josef Deutscher

France

Yvan Devaux

Luxembourg

Zoltan Dezso

USA

Swatismita Dhar

India

Sangeeta Dhaubhadel

Canada

Amit Dhingra

USA

Graciela Dias

Brazil

Norbeto Díaz-Díaz

Spain

Walburga Dieterich

Germany

Stephen Difazio

USA

Lubbert Dijkhuizen

Netherlands

Johannes Dijkstra

Japan

Rebecca Dikow

USA

Rod Dimaline

UK

David Dineen

Ireland

Lianghao Ding

USA

Randy Dinkins

USA

Jonathan Dinman

USA

Christian Diot

France

Simon Dittami

Norway

Dirk Dittmer

USA

Marcus Dittrich

Germany

Michael Djordjevic

Australia

Dirk Dobbelaere

Switzerland

Anna Dobritsa

USA

Harsha Vardhan Doddapaneni

USA

Eric Dodds

USA

Jerry Dodgson

USA

Marlies Dolezal

Austria

Jaroslav Dolezel

Czech Republic

Lars Dölken

UK

Eytan Domany

Israel

Janet Donaldson

USA

Claudio Donati

Italy

Zheng Dong

USA

Nilgun Donmez

Canada

Giuseppe D'Onofrio

Italy

Scott Dougan

USA

Robin Dowell

USA

Finn Drabløs

Norway

Milenkovic Dragan

France

Pavel Drevinek

Czech Republic

Philipp Drewe

Germany

Christine Dreyer

Germany

Jean-Michel Drezen

France

Patrick D'Silva

India

Ursula D'Souza

UK

Lixin Du

China

Charles Du

USA

Yongping Duan

USA

Christian Dubos

France

Christele Dubourg

France

Jean Dubremetz

France

Michel Duclos

France

Joel Dudley

USA

Alexandra Dumitriu

USA

Beth Dumont

USA

Susana Dunner

Spain

Sébastien Duplessis

France

Bas E. Dutilh

Netherlands

Chitra Dutta

India

Jan Dvorak

USA

David Dyer

USA

Paul Dyson

UK

Andrew Eamens

Australia

Karen Echeverri

USA

Craig Echt

USA

Andrew Eckert

USA

Inger Edfors

Sweden

Christine Edwards

USA

Owain Edwards

Australia

Juergen Ehlting

Canada

Patrick Eichenberger

USA

John Eimes

USA

Christer Einvik

Norway

Ulrich Lothar Maria Eisel

Netherlands

Robert Ekblom

Sweden

Martin Elferink

Netherlands

Hans Ellegren

Sweden

Thomas Henry Noel Ellis

UK

Peter Ellis

UK

Paula Elomaa

Finland

Najib El-Sayed

USA

Mostafa Elshahed

USA

Abdou Elsharawy

Germany

Christine Elsik

USA

Bert Ely

USA

Scott Emrich

USA

Uli Ernst

Belgium

Patricia Ernst

USA

Matthew Escobar

USA

Daniel Esmenjaud

France

Anna Esteve-Codina

Spain

Luke Evans

USA

Helen Evans

UK

Oystein Evensen

Norway

Alexei Evsikov

USA

Eduardo Eyras

Spain

Tariq Ezaz

Australia

Hiroshi Ezura

Japan

Foucher Fabrice

France

Marc Facciotti

USA

Luigi Faino

Netherlands

Patricia Faivre Rampant

France

Ahmad Fakhoury

USA

Bin Fan

China

Jian-Bing Fan

USA

David Fang

USA

Jinggui Fang

China

Marisa Farber

Argentina

Mario Fares

Spain

Rhys Farrer

UK

Mark Fast

Canada

Melanie Febrer

UK

Natalie Fedorova Abrams

USA

Zhangjun Fei

USA

Robert Feil

France

Mark Feinberg

USA

Heike Feldhaar

Germany

Barbara Feldmeyer

Germany

John Fellers

USA

Malcolm Ferguson-Smith

UK

Jorge Fernandes

Norway

Cathy Fernandes

UK

Alisdair Fernie

Germany

Jennifer Fettweis

USA

Peter Fields

USA

Agnes Figueiredo

Brazil

Maria Figueroa

USA

Matthias Fischer

Germany

Andre Fischer

Germany

Gilles Fischer

France

Jason Fish

Canada

Susan Fisher

USA

J Ross Fitzgerald

UK

Laurence Flori

France

Josep M Folch

Spain

Jessica Fong

USA

Paolo Fontana

Italy

Luca Fontanesi

Italy

Sylvain Forêt

Australia

Javier Forment

Spain

Eduardo Formighieri

Brazil

Kristoffer Forslund

Germany

John Forster

Australia

Konrad Ulrich Förstner

Germany

Caroline Fossum

Sweden

Gerda Fourie

South Africa

Florence Frager

France

Christof Francke

Netherlands

Andre Franke

Germany

Adam Frankish

UK

Elisabetta Frascaroli

Italy

Pina Fratamico

USA

Erik Fredlund

Sweden

David Fredman

Austria

Brad Freking

USA

Leon French

Canada

W. Florian Fricke

USA

Swetlana Friedel

Germany

Marc Friedländer

Spain

Thomas Friedman

USA

Jason Fritzler

USA

Hillel Fromm

Israel

Florian Frugier

France

USA

Andrew Fry

UK

Bryan Fry

Australia

Rebecca Fry

Karl Fryxell

USA

Jinmin Fu

China

Heiko Fuchs

Germany

Masaya Fujita

USA

Yasutaro Fujita

Japan

Ricardo Fujiwara

Brazil

Shoko Fujiwara

Japan

Melissa Fullwood

Singapore

Jaroslav Fulnecek

Czech Republic

Noriko Funato

Japan

Ryo Futahashi

Japan

Agata Gadaleta

Italy

Carlo Gaetano

Germany

Nellie Gagne

Canada

Julien Gagneur

Germany

Albert Gahr

USA

Carlos Galeano

Belgium

Juan Galindo

Spain

Giulio Galla

Italy

Irene Gallego Romero

UK

Michael Galperin

USA

Eric Gamazon

USA

Anand Ganesan

USA

Eric Ganko

USA

Victor Gannon

Canada

Steve Gantt

USA

Jean Gao

USA

Jianjiong Gao

USA

Javier Garaizar

Spain

Tzintzuni Garcia

USA

Juan Antonio Garcia

Austria

Elsa Garcia-Gamez

Spain

Maria Rosario Garcia-Gil

Sweden

Jose Manuel Garcia-Manteiga

Italy

Jordi Garcia-Mas

Spain

Ramón García-Sanz

Spain

Mike Gardner

Australia

Richard Gardner

New Zealand

Malcolm Gardner

USA

George Garrity

USA

Daniel Garza

Brazil

Susan M Gasser

Switzerland

Mathieu Gautier

France

Virginie Wilhelmine Gautier

Ireland

Joel Gautron

France

Isabella Gazzoli

Netherlands

Xuejun Ge

China

Melaku Gedil

Nigeria

Mikhail Gelfand

Russian Federation

Vincenzo Alessandro Gennarino

USA

Marila Gennaro

USA

Laura Genovesi

Australia

Alessandra Gentile

Italy

Laurent Gentzbittel

France

Paul Gepts

USA

Nicole Gerardo

USA

Anna Gerasimova

USA

Daniel Gerlach

Austria

Marcelo German

USA

Johannes Gescher

Germany

Ad Geurts Van Kessel

Netherlands

Matthias Geyer

Germany

Shanaz Ghandhi

USA

Murad Ghanim

Israel

Godelieve Gheysen

Belgium

Othman Ghribi

USA

Carmen Gianfrani

Italy

Daniel Gianola

USA

Emiliano Giardina

Italy

Joshua Gibson

USA

Mark Gijzen

Canada

Rosario Gil

Spain

Daniel Gilbert

Germany

Jason Gill

USA

Joseph Gillespie

USA

Jeffrey Gimble

USA

Michael Ginger

UK

Hila Gingold

Israel

Jean-Marc Gion

France

Antonio Giordano

USA

Federico Giorgi

Italy

Eric Giraud

France

Matt Gitzendanner

USA

Elisabetta Giuffra

Italy

Giovanni Giuliano

Italy

Tibor Glant

USA

Gernot Gloeckner

Germany

Philip Goetz

USA

Maria Helena Goldman

Brazil

Katsuya Gomi

Japan

Florence Gondret

France

Cedric Gondro

Australia

Zhiyuan Gong

Singapore

Martine Gonneau

France

Alison Goodall

UK

Michele Goodhardt

France

Francoise Gosti

France

Dietrich Gotzek

USA

Julian Gough

UK

Aleksander Grabiec

Netherlands

David Grant

USA

Murray Grant

UK

Dario Grattapaglia

Brazil

Boris Greber

Germany

Catherine Greene

Ireland

Casey Greene

USA

William Greenleaf

USA

Vivi Raundahl Gregersen

Denmark

Brian Gregory

USA

Ruth Grene

USA

Malachi Griffith

USA

Andrew Griffiths

New Zealand

Sam Griffiths-Jones

UK

Igor Grigoriev

USA

Johannes Grillari

Austria

Jacqueline Grima-Pettenati

France

Unni Grimholt

Norway

Nigel Harry Grimsley

France

Martien Groenen

Netherlands

Andrew Groover

USA

Joshua Gross

USA

Robert Grosse

Germany

Arthur Gruber

Brazil

Aleksandra Gruca

Poland

Kristina Gruden

Slovenia

Rebecca Grumet

USA

Christoph Grunau

France

Frank Grutzner

Australia

Zhenglong Gu

USA

Xingyou Gu

USA

Jianying Gu

USA

Rongzhan Guan

China

Chittibabu Guda

USA

Yannick Gueguen

France

Felix Guerrero

USA

Nancy Guillen

France

Patricia M Guimaraes

Brazil

Wangzhen Guo

China

Baozhu Guo

USA

Wen-Wu Guo

China

Zhenhua Guo

China

Ramneek Gupta

Denmark

Radhey Gupta

Canada

Michael Gurevitz

Israel

Priyatansh Gurha

USA

Santiago Gutierrez

Spain

Alfonso Gutierrez-Adan

Spain

Lionel Guy

Sweden

René Guyomard

France

Carlos Guzman

Spain

Vladimir Gvozdev

Russian Federation

Christoph Haag

Switzerland

Brian Haas

USA

Rainer Haas

Germany

Yoshiki Habu

Japan

Michael Hackenberg

Spain

Hubert Hackl

Austria

Stéphane Hacquard

France

Daniel Haft

USA

Matthew Hahn

USA

Stephan Hahn

Germany

Hu Haiyang

China

Anders Hakansson

USA

Mohamed-Ali Hakimi

France

Matthew Hale

USA

Chris Haley

UK

Marc Halfon

USA

Jorg Hamann

Netherlands

Ann Hammonds

USA

Jochen Hampe

Germany

Leng Han

USA

Jae Han

Korea, South

Hirokazu Handa

Japan

Alfred Handler

USA

Helmut Hanenberg

USA

Anthony Hannan

Australia

Sridhar Hannenhalli

USA

Lars Hestbjerg Hansen

Denmark

Weilong Hao

USA

Thomas James Hardcastle

UK

Stephen Hardies

USA

Barbara Harlizius

Netherlands

Luke Harmon

USA

Andrea Harper

UK

Matthew Harris

USA

Paul Harrison

Canada

Robert Harrison

USA

Edgar Hartsuiker

UK

Simon Harvey

UK

Masato Hasegawa

Japan

Koji Hasegawa

Japan

Robert Haselkorn

USA

Grit Haseneyer

Germany

Ros Hastings

UK

Graham Hatfull

USA

Naoko Hattori

Japan

Loren Hauser

USA

Michael Havey

USA

Vitezslav Havlicek

Austria

David Hawkins

USA

Jennifer Hawkins

USA

Johannes Haybaeck

Austria

Kevin Hayes

USA

Alice Hayward

Australia

Dong He

USA

Guohao He

USA

Ping-An He

China

Yuehui He

Singapore

Denis Headon

UK

Edith Heard

France

Simon Heath

Spain

Joshua Heazlewood

USA

Julia Heck

USA

Jakob Hedegaard

Denmark

Mohammad Heidari

USA

Jörg Heierhorst

Australia

Juergen Heinisch

Germany

Mark Heise

USA

Chris Helliwell

Australia

Asaf Hellman

Israel

Prasad Hendre

India

Steven Henikoff

USA

Rc Hennekam

Netherlands

Michael Hensel

Germany

David Henshall

Ireland

John Henshall

Australia

Rosa Hermosa

Spain

Amaury Herpin

Germany

Jeremy Herren

Switzerland

Ralf Herwig

Germany

Wolfgang Rene Hess

Germany

Robert Hettich

USA

Kim Heylen

Belgium

Stefan Hiendleder

Australia

Catherine Hill

USA

Emmeline Hill

Ireland

Beate Hiller

Germany

Dirk Hincha

Germany

David Hinton

USA

William Hintz

Canada

Christophe Hitte

France

Chris Todd Hittinger

USA

Robert Hitzemann

USA

Oliver Hobert

USA

Charlie Hodgman

UK

Peter Hodson

Canada

Federico Guillermo Hoffmann

USA

Amber Hogart Begtrup

USA

John Hogenesch

USA

C. Corley Holbrook

USA

Jason Holliday

USA

Ed Hollox

UK

Rikard Holmdahl

Sweden

Roger Holmes

USA

Thomas Holstein

Germany

Kathryn Holt

Australia

Yunhan Hong

Singapore

Guido Hooiveld

Netherlands

Tiago Hori

Canada

Eran Hornstein

Israel

Csaba Hornyik

UK

Paul Horrocks

UK

David Horvath

USA

Steve Horvath

USA

Benjamin A. Horwitz

Israel

Roger Hoskins

USA

Ross Houston

UK

Glenn Howe

USA

Pei-Yin Hsu

USA

Jinguo Hu

USA

Zihua Hu

USA

Zhihua Hua

USA

Bevan Emma Huang

Australia

Dawei Huang

USA

Hao-Jen Huang

Taiwan

Hsien-Da Huang

Taiwan

Hsuan-Cheng Huang

Taiwan

Hui Huang

USA

Jiaoti Huan

USA

Jingshan Huang

USA

R. Stephanie Huang

USA

Shaobai Huang

Australia

Tinghua Huang

China

Wen Huang

USA

Wendong Huang

USA

Yongping Huang

China

Nicholas James Hudson

Australia

Austin Hughes

USA

Timothy Hughes

Canada

Scot Hulbert

USA

Amanda Hummon

USA

Arthur Hunt

USA

Brendan Hunt

USA

Peter Hunt

Australia

José Hureaux

France

Klaus Huse

Germany

Curtis Huttenhower

USA

Michael Hynes

Australia

Timo Hytönen

Finland

Eveline Ibeagha-Awemu

Canada

Antti Iivanainen

Finland

Tohru Ikuta

USA

Aaron Ingham

Australia

Jun Inoue

Japan

Ilya Ioshikhes

Canada

Manuel Irimia

Canada

Erin Irish

USA

Fumitoshi Ishino

Japan

Sachiko Isobe

Japan

Mayumi Ito

USA

Takeshi Itoh

Japan

Thomas Jack

USA

Shaun Jackman

Canada

Andrew Paul Jackson

UK

Mike Jackson

UK

Robert Jackson

UK

Scott Jackson

USA

Kevin Jacobs

USA

George Jacoby

USA

Guru Jagadeeswaran

USA

Mukesh Jain

India

Andrew James

Mexico

Luc Janss

Denmark

Julie Jaquiery

France

Stephanie Jaubert-Possamai

France

Kuan-Teh Jeang

USA

Aaron Jeffries

UK

Kerstin Jekosch

UK

Deborah Jenson

USA

Loydie Jerome-Majewska

Canada

Aaron Jex

Australia

Ping Ji

USA

Guoli Ji

China

Yuan Ji

USA

Yuanqing Jiag

China

Li Jia-Le

China

Cizhong Jiang

China

Lichun Jiang

USA

Lixi Jiang

China

Yiwei Jiang

USA

Meilin Jin

China

Marek Jindra

Czech Republic

Hai-Chun Jing

China

Huaiqi Jing

China

Alan L. Johnson

USA

Greg Johnson

USA

Jerald B. Johnson

USA

Louise Johnson

UK

Patricia Johnson

USA

Zach Johnson

USA

Alex Jones

UK

Gary Jones

Ireland

Michael Jones

UK

Neil Jones

UK

Matthew Jones-Rhoades

USA

Yuh-Shan Jou

Taiwan

Paula Jouhten

Finland

Klaus Jung

Germany

Marc Jung

USA

James Jurgenson

USA

Art K

USA

Haja Kadarmideen

Denmark

Shawn Kaeppler

USA

Uzi Kafkafi

Israel

Daniel Kahn

France

Kriton Kalantidis

Greece

Alex Thomas Kalinka

Germany

Paul Kalitsis

Australia

Birgitte H. Kallipolitis

Denmark

Thomas Källman

Sweden

Allan Kalueff

USA

Yoichiro Kamatani

France

Eli Kaminuma

Japan

Sophien Kamoun

UK

Shigehiko Kanaya

Japan

Eugene Kandel

USA

Angelos Kanellis

Greece

Zhensheng Kang

China

Le Kang

China

Aditi Kanhere

UK

Ronan Kapetanovic

UK

Emre Karakoc

USA

Genesio Karere

USA

Magnus Karlsson

Sweden

Khalil Kashkush

Israel

Sophia Kathariou

USA

Santosh Katiyar

USA

Michael G Katze

USA

Jim Kaufman

UK

Rakesh Kaundal

USA

Julia Kehr

Spain

Yogeshwar Kelkar

USA

Colin Kelleher

Ireland

Andreas Keller

USA

John M Kelly

UK

Libusha Kelly

USA

Kathryn Kemper

Australia

Frank Kempken

Germany

Stephen Kent

Australia

Paul Kersey

UK

Birgit Kersten

Germany

Hindrik Harm Dirk Kerstens

Netherlands

Philipp Khaitovich

Germany

Raya Khanin

USA

Mehar S Khatkar

Australia

Jeffrey Kidd

USA

Mark Kidd

USA

James Wraith Kijas

Australia

Shoshi Kikuchi

Japan

Mogens Kilian

Denmark

Andrzej Kilian

Australia

Duk Kyung Kim

USA

Sanghyeon Kim

USA

Sangsoo Kim

Korea, South

Yongsig Kim

USA

Tjeerd Kimman

Netherlands

Shioko Kimura

USA

Martin Kircher

Germany

Matias Kirst

USA

Konstantin Kiselev

Russian Federation

Uday Kishore

UK

Jacob Kitzman

USA

Eline Klaassens

Australia

Eric Klee

USA

Daniel Kliebenstein

USA

Carolyn Klinge

USA

Robert Klopfleisch

Germany

Brian Knights

United States Minor Outlying Islands

Volker Knoop

Germany

Takato Koba

Japan

Takehiko Kobayashi

Japan

Thomas Kocher

USA

Josef Koeck

Germany

Michael Kogut

USA

Ho-Jin Koh

USA

Annegret Kohler

France

Joshua Kohn

USA

Takashi Kohno

Japan

Kenji Kojima

USA

Sulev Koks

Estonia

Catherine Kolditz

France

Anne-Brit Kolsto

Norway

Xiangyin Kong

China

Kostas Konstantinidiss

USA

David Kopecky

Czech Republic

Andy Koppisch

USA

Ulrike Korf

Germany

Helena Korpelainen

Finland

Hajime Kosaka

Japan

Arkadiusz Kosmala

Poland

Susan Koval

Canada

Mallikarjuna Rao Kovi

Norway

Christine Kozak

USA

Piotr Kozlowski

Poland

Dmitri Kramerov

Russian Federation

Sven Krappmann

Germany

Galina Kravatskaya

Russian Federation

Lukas Krebes

Germany

David P. Kreil

Austria

Markus Kreuz

Germany

Jeroen Krijgsveld

Germany

Annangarachari Krishnamachari

India

Neeraja Krishnan

India

David Kristensen

USA

Anne Krogsdam

Austria

Andrew Kropinski

Canada

Marija Krstic-Demonacos

UK

Tomohiko Kubo

Japan

Indira Kudva

USA

Hao Kueh

USA

Ursula Kües

Germany

Heiner Kuhl

Germany

Anna Kukekova

USA

Vanaja Kumar

India

Yoshinori Kumazawa

Japan

Ruprecht Kuner

Germany

Axel Künstner

Germany

Reinhard Kunze

Germany

Chih-Horng Kuo

Taiwan

Diederik Kuster

USA

Vladimir Kuznetsov

Singapore

Dan Kvitek

USA

Jean Labarre

France

Kaitao Lai

Australia

Eric Lai

USA

Yinglei Lai

USA

Marcelo Laia

Brazil

Jonathan Lamarre

Canada

David Lambert

Australia

David Landsman

USA

Gregory Lang

USA

Mette Langaas

Norway

Nicolas Langlade

France

Richard Lapoint

USA

John Lapres

USA

Michael Lardelli

Australia

Pär Larsson

Sweden

Tomas Larsson

Germany

Edwin Lasonder

Netherlands

Howard Laten

USA

Mario Latendresse

USA

Audrey Lau

USA

Patrick Laufs

France

Louise Laurent

USA

Enrico Lavezzo

Italy

Martin Lavin

Australia

Jeffrey Lawrence

USA

Alice Layton

USA

Barbara Lazzari

Italy

Antony Le Béchec

Luxembourg

Fabienne Le Provost

France

Frederique Le Roux

France

Gael Le Trionnaire

France

Jared Leadbetter

USA

Rebecca Leary

USA

Erica Leder

Finland

Chia Lee

USA

Daeyoup Lee

Korea, South

Dong-Yup Lee

Singapore

Hane Lee

USA

Jong Eun Lee

Korea, South

Ruey-Hua Lee

Taiwan

Suk-Ha Lee

Korea, South

Christophe Lefevre

Australia

Philippe Lefrançois

USA

Fabrice Legeai

France

Matthieu Legendre

France

Ulrich Lehmann

Germany

Sigrid Lehnert

Australia

Elissa Lei

USA

Andrew Leitch

UK

Ed Leiter

USA

Brigitte Lelongt

France

Giorgio Lenaz

Italy

Tobias Lenz

USA

Emmanuelle Lerat

France

Christine Leroux

France

Anthony Leung

USA

Erez Levanon

Israel

Nils Anders Leversen

Norway

Andre Levesque

Canada

Amnon Levi

USA

Henry Levin

USA

Mia Levine

USA

Avraham Levy

Israel

Ala Lew-Tabor

Australia

Bailin Li

USA

Chuan-Yun Li

China

Jianjun Li

China

Jie Li

Germany

Jin Billy Li

USA

Lang Li

USA

Lei Li

Canada

Lei Li

USA

Leping Li

USA

Ming Li

USA

Mingyao Li

USA

Shawn Li

Canada

Sheng Li

China

Weizhe Li

USA

Weizhong Li

USA

Wenbin Li

China

Xinguo Li

Australia

Xuewei Li

China

Yudong Li

China

Ping Liang

Canada

Chun Liang

USA

Ben-Yang Liao

Taiwan

Laurence Liaubet

France

Alan Wee-Chung Liew

Australia

David Lightfoot

USA

Han Lim

USA

Yong Pyo Lim

Korea, South

Chan-Shing Lin

Taiwan

Ching-Ting Lin

Taiwan

Lei Lin

China

Wen-Chang Lin

Taiwan

Zhenguo Lin

USA

Steven Lindow

USA

Arimantas Lionikas

UK

Damon Lisch

USA

Aaron Liston

USA

George Liu

USA

G Y Liu

China

Guozhen Liu

China

Jianquan Liu

China

John Liu

USA

Kede Liu

China

Renyi Liu

USA

Shikai Liu

USA

Shu-Lin Liu

Canada

Tao Liu

China

Xiling Liu

China

Yin Liu

USA

Zhandong Liu

USA

Zhi-Ping Liu

China

Jonathan Livny

USA

Ana Llopart

USA

Neil Lobo

USA

Helen Lockstone

UK

Manyuan Long

USA

Juan Loor

USA

Joyce Loper

USA

Ana-Flor Lopez-Millan

Spain

Aaron Lorenz

USA

Cheng Lu

USA

Yubing Lu

USA

Zhi Lu

China

Francesca Luca

USA

Reini Luco

France

Adam Lukaszewski

USA

Walter Lukiw

USA

Steven Lund

Canada

Katie Lunnon

UK

Feng Luo

USA

Zewei Luo

UK

Volodymyr Lushchak

Ukraine

Carol Lutz

USA

Minh Cuc Luu

Viet Nam

Frank Lyko

Germany

David Lynn

Ireland

Peilin Ma

USA

Xiaotu Ma

USA

Yibao Ma

China

Youzhi Ma

China

Avi Ma'Ayan

USA

Robert L. Mach

Austria

Carlos Machado

USA

John Mackay

Canada

Emilia Madarász

Hungary

pni Madasamy

India

Andras Madi

Hungary

Ole Madsen

Netherlands

Jeroen Maertzdorf

Germany

Reedik Mägi

Estonia

Rohit Mago

Australia

Nancy Mah

Germany

Ramamurthy Mahalingam

USA

Alexis Maizel

Germany

Sadhan Majumder

USA

Kira Makarova

USA

Vsevolod Makeev

Russian Federation

Giovanni Malerba

Italy

Bonnie Mallard

Canada

Mickael Malnoy

Italy

Boris Malyarchuk

Russian Federation

Eugenio Mancera

USA

Mauro Mandrioli

Italy

Elisabetta Manduchi

USA

Karlheinz Mann

Germany

Ulrich Mansmann

Germany

Roberto Mantovani

Italy

Nitin Mantri

Australia

Long Mao

China

Nauman Maqbool

New Zealand

Antonio Marco

UK

Helmi Mardassi

Tunisia

Jim Marden

USA

Frantisek Marec

Czech Republic

Michael Mares

Czech Republic

Rogerio Margis

Brazil

Tomislav Maricic

Germany

Ignacio Marin

Spain

Adrian Mariño-Enriquez

USA

Leonardo Marino-Ramirez

USA

Osvaldo Marinotti

USA

Victor Markowitz

USA

Jeffrey Marks

USA

Rolf Marschalek

Germany

David Marshall

UK

John Martens

Netherlands

Andrew Martin

USA

Lee Martin

USA

Pascal Martin

France

Sam Martin

UK

Jesus Martinez-Barnetche

Mexico

Esperanza Martinez-Romero

Mexico

Emmanuelle Martini

France

Cesar Martins

Brazil

Isabelle Martin-Verstraete

France

Maryann Martone

USA

Shinichiro Maruyama

Canada

Lebrun Maryse

France

Joanna Masel

USA

Pascal Maser

Switzerland

John P Masly

USA

Annaliese Mason

Australia

Christopher Mason

USA

Ali Masoudi-Nejad

Iran

Emma Master

Canada

Anna Maria Mastrangelo

Italy

Juan Mata

UK

Antonio J Matas Arroyo

USA

Dante Mateo

Canada

Sarah Mathews

USA

Sachihiro Matsunaga

Japan

Shigenobu Matsuzaki

Japan

Lakshmi Matukumalli

USA

Mikhail Matz

USA

Julie Maupin-Furlow

USA

David Mazurais

France

Matthew Nicholson Mccall

USA

Fiona Mccarthy

USA

Phillip Mcclean

USA

Matthew Mcclure

USA

John Mccusker

USA

Tara Mcdaneld

USA

Beatrice Mcgivney

Ireland

Michael Mcinerney

USA

Stephanie Mckay

USA

Aoife Mclysaght

Ireland

Shannon Mcweeney

USA

Kieran Meade

Ireland

Vivien Measday

Canada

Françoise Médale

France

Mario Medvedovic

USA

Karyn Megy

UK

Michael Mehan

USA

Angela Mehta

Brazil

Annemarie Meijer

Netherlands

Sebastiaan Meijsing

Germany

Steven Meinhardt

USA

Lee Meisel

Chile

Claudio Mello

USA

Erdogan Memili

USA

Daniel Menendez

USA

Wieslawa Mentzen

Italy

Ralph Menzel

Germany

Uwe Menzel

Germany

Shannath Merbs

USA

Angelika Merkel

Japan

Eran Meshorer

Israel

Cornelia Metges

Germany

Barbara Methe

USA

Axel Meyer

Germany

Eli Meyer

USA

Folker Meyer

USA

Andreas Meyerhans

Spain

Blake Meyers

USA

Jason Mezey

USA

Erica Mica

Italy

Shelly Michalski

USA

Andy Michel

USA

Diego Micheletti

Spain

Jonathan Mielenz

USA

Lili Milani

Estonia

Pawel Milczarski

Poland

Jeremy Miles

USA

Jonathan Mill

UK

Tony Millar

Australia

Christopher Miller

USA

Eric Miller

USA

Jason Miller

USA

Jeremy Miller

USA

Kristi Miller

Canada

Michael Miller

USA

Robert Miller

USA

David Mills

USA

Xiangjia Jack Min

USA

Wongi Min

Korea, South

Yuki Minegishi

Netherlands

Ray Ming

USA

Dominique Mingeot

Belgium

Alex Mira

Spain

Juan Miranda-Rios

Mexico

Alexandre Mironov

Italy

Caterina Missero

Italy

Ignacy Misztal

USA

Kevin Mitchell

Ireland

Phil Mitchell

UK

Robert Mitchell

USA

Rowan A C Mitchell

UK

Wilfrid Mitchell

UK

Omprakash Mittapalli

USA

Tohru Miyoshi-Akiyama

Japan

Eshchar Mizrachi

South Africa

Kenji Mizuguchi

UK

Kenji Mizuguchi

Japan

Ralf Moeller

Germany

Thomas Moen

Norway

Cecilia Moens

USA

Perry Moerland

Netherlands

Simone Moertl

Germany

Soren Moestrup

Denmark

Jeremy Mogridge

Canada

Trilochan Mohapatra

India

Igor Mokrousov

Russian Federation

Adrian Molenaar

New Zealand

James Monaghan

USA

Amparo Monfort

Spain

Emmanuel F. Mongodin

USA

Michel Monod

Switzerland

Paula Maria Moolhuijzen

Australia

Ramal Moonesinghe

USA

Robert Moore

Australia

Lucia Morales

France

Maria Morales

USA

Ana Paula B. Moreira

Brazil

Natalia Moreno-Sanchez

Spain

Marco Moretto

Italy

Richard Morgan

USA

John Morgan

USA

Paul Morris

USA

Hilary Morrison

USA

Mirko Moser

Italy

Mary Motherway

Ireland

Kazushi Motomura

Japan

Mario Motto

Italy

Raphael Mourad

USA

Gabriel Mourente

Spain

Scott Moye-Rowley

USA

Julien Mozziconacci

France

Jianbing Mu

USA

Mauricio Mudadu

Brazil

Jonathan Mudge

UK

Lukas Mueller

USA

Chandra Mukhopadhyay

India

Subhasis Mukhopadhyay

India

Victoriano Mulero

Spain

Jeong-Hwan Mun

Korea, South

Maite Muniesa

Spain

Sarah Munro

USA

Chris Murgatroyd

Germany

Antonio Musio

Italy

Garry Myers

USA

Peter Myler

USA

Satoshi Nagai

Japan

Takeshi Nagashima

Japan

Laszlo Nagy

Hungary

Sven Nahnsen

Germany

Shinichi Nakagawa

Japan

Kinichi Nakashima

Japan

Harikrishna Nakshatri

USA

Satoshi Namekawa

USA

Bindu Nanduri

USA

Loris Nanni

Italy

Alfonso Navas

Spain

Patrick Navas

USA

Frank Naya

USA

Hugo Naya

Uruguay

Daniel Neafsey

USA

David Neale

USA

Aurora Nedelcu

Canada

Uwe Nehls

Germany

David Nelson

USA

George Nelson

USA

Karen Nelson

USA

Matthew Nelson

Australia

Mitsuru Nenoi

Japan

Rob Ness

UK

Annette Neubüser

Germany

Cécile Neuvéglise

France

Pierre Neuvial

France

Cedric Neveu

France

Margaret Neville

USA

Henry Nguyen

USA

Kwangsik Nho

USA

Francesco Nicassio

Italy

Krista Nichols

USA

Rick Nicholson

Australia

Marisa F Nicolás

Brazil

Oliver Niehuis

Germany

Henrik Nielsen

Denmark

Kay Nieselt

Germany

Carien Niessen

Germany

Sergey Nikolaev

Switzerland

Mats Nilsson

Sweden

Hidenori Nishihara

Japan

Chikako Nishitani

Japan

Eishi Noguchi

USA

Fabio Nogueira

Brazil

Hisayuki Nomiyama

Japan

Dan Nonneman

USA

Joseph Norman

Canada

Mehrdad Noruzinia

Iran

Michael Nothnagel

Germany

Masafumi Nozawa

Japan

Alexis Nzila

Kenya

Ulrike Ober

Germany

David Oconnor

USA

Naotake Ogasawara

Japan

Norichika Ogata

Japan

Yoshitoshi Ogura

Japan

Terrance O'Hanlon

USA

Tazro Ohta

Japan

Miki Okada

USA

Shigeru Okada

Japan

Mina Okochi

Japan

Michal Okoniewski

Switzerland

Carla Oliveira

Portugal

Patrícia Oliveira

Portugal

Jose L. Oliver

Spain

Patrick Ollitrault

France

Ingrid Olsaker

Norway

Daniel Olson

USA

Bode Olukolu

USA

Seong Siang Ong

Malaysia

Charles Opperman

USA

Monica Orellana

USA

Aharon Oren

Israel

Gisella Orjeda

Peru

Ernesto Ormeno-Orrillo

Mexico

Naoki Osada

Japan

Cameron Osborne

UK

Nir Osherov

Israel

Carla Osiowy

Canada

Majid Osman

Sweden

Justin O'Sullivan

New Zealand

Martin Oti

Netherlands

Nicholas O'Toole

Canada

Sieglinde Ott

Germany

Hong-Yu Ou

China

Marc Ouellette

Canada

Yoshihiro Ozeki

Japan

Fatih Ozsolak

USA

Amanda Padovan

Australia

Mark Paine

UK

Jorge Paiva

Portugal

Suman Pakala

USA

Sharmistha Pal

USA

Irene Pala

Sweden

Dasaradhi Palakodeti

India

Stela Palii

USA

Lance Palmer

USA

Yniv Palti

USA

Franck Panabieres

France

Ashwni Kumar Pandey

India

Girdhar Pandey

India

Nattanan Panjaworayan

Thailand

Georgia Panopoulou

Germany

Stéphane Panserat

France

Ilan pn

Israel

Isobel Parkin

Canada

Matthew Parks

USA

Larry Parnell

USA

Zuly Parra

USA

John Parsch

Germany

Michael James Parsons

UK

Sara Partemi

Italy

Erica Pasini

Netherlands

Andrew Pask

USA

Karla Passalacqua

USA

Flavia Passos

Brazil

Prabhu Patil

India

Yannick Pauchet

Germany

Nathalie Pavy

Canada

Gary Payne

USA

Terry Pearson

Canada

Inti Pedroso

UK

Francisco Pelegri

USA

Pablo Pelegrin

Spain

Matteo Pellegrini

USA

Marina Peluffo

Argentina

Francisco Peñagaricano

USA

Luiz Penalva

USA

Miguel Penalva

Spain

Michele Perazzolli

Italy

Andy Pereira

USA

Ernesto Perez-Rueda

Mexico

George Perry

USA

Mark Perry

UK

Graziano Pesole

Italy

Elena Pestsova

Germany

Sunday Peters

USA

Kevin Peterson

USA

Tobias Hartmut Petri

Germany

Sandrine Petry

France

Jonathan Pevsner

USA

Belen Pico

Spain

Stefania Pilati

Italy

Lori Pile

USA

Madalena Pimentel

Portugal

Stefan Pinter

USA

Paulo Marcos Pinto

Brazil

Rosa Maria Pintó

Spain

Helen Piontkivska

USA

Roger Pique-Regi

USA

J. Chris Pires

USA

Nuno Pires

Switzerland

Josep V. Planas

Spain

Graham Plastow

Canada

Federico Plazzi

Italy

Karine Plourde

Canada

Olga I. Podgornaya

Russian Federation

Monica Poelchau

USA

R. Scott Poethig

USA

Igor Pogribny

USA

Julio Polaina

Spain

Eugenia Poliakov

USA

Nicolas Pollet

France

Andrew Pomiankowski

UK

Igor Ponomarev

USA

Siriluck Ponsuksili

Germany

Beatriz Pontes

Spain

Leo Poon

Hong Kong

Ron Porat

Israel

Alexey Porollo

USA

Ezio Portis

Italy

Charlotte Poschenrieder

Spain

David Pot

USA

Deborah Power

Portugal

Rolf Prade

USA

Sylvain Pradervand

Switzerland

Ashwin Prakash

USA

Manoj Prasad

India

Shelly Praveen

India

Thomas Preiss

Australia

Michael Prentice

Ireland

Silvano Presciuttini

Italy

James Preston

USA

Julia Pridgeon

USA

Alessandro Prigione

Germany

Michael Primig

France

Andreas Prokesch

Austria

Nicholas Provart

Canada

Anna Psaroulaki

Greece

Soni Savai Pullamsetti

Germany

Marco Punta

UK

Mirja Puolakkainen

Finland

Luciano Puzer

Brazil

Joshua Puzey

USA

Ji Qi

China

Huaizhen Qin

USA

Li-Xuan Qin

USA

Deyou Qiu

China

Peng Qiu

USA

Kun Qu

USA

Nicole Quinn

Canada

Fabiola Quintero-Rivera

USA

Petr Ráb

Czech Republic

Monte Radeke

USA

Hermann Ragg

Germany

Johannes Rainer

Austria

Istvan Rajcan

Canada

Markus Ralser

UK

Srinivasan Ramachandran

India

Sahadevan Raman

USA

Karthik Raman

India

Mani Ramaswami

Ireland

Mika Ramet

Finland

Jean-Francois Rami

France

Punna Ramu

India

David Rand

USA

A. Koneti Rao

USA

Aakrosh Ratan

USA

Narayan Rath

USA

Thomas Rattei

Austria

Dmitry Ravcheev

Russian Federation

David Ray

USA

Timothy Read

USA

Ahmed Rebai

Tunisia

Venu Reddyvari Channarayappa

USA

John Reece-Hoyes

USA

James Reecy

USA

Kent Reed

USA

Sharon Regan

Canada

Gemma Reguera

USA

Michael Rehli

Germany

Adam Reid

UK

Gregor Reid

Canada

Norbert Reiling

Germany

Mark Reimers

USA

Valerie Reinke

USA

Bernhard Renard

Germany

Carlo Renieri

Italy

Susanne Renner

Germany

Pierre-Yves Rescan

France

Wolfgang Resch

USA

Caird Rexroad

USA

Jose Ribeiro

USA

Anne Ricard

France

Antonella Riccio

UK

Jose Luis Riechmann

USA

Jose Luis Riechmann

Spain

Katharina Riedel

Germany

Guillem Rigaill

France

Monique Rijnkels

USA

Robert Riley

USA

Gerald Rimbach

Germany

Gonzalo Rincon

USA

Brian Ring

USA

Silke Rinkwitz

Australia

Matthew Rise

Canada

Claude Rispe

France

Davide Risso

USA

Matthew Ritchie

Australia

Kermit Ritland

Canada

Rita Roberti

Italy

Jeremy Roberts

UK

Philip Roberts

USA

Ashley Robinson

USA

Eduardo Rocha

France

Mauricio Rodriguez-Lanetty

USA

Susana Rodriguez-Navarro

Spain

Francisco Rodriguez-Valera

Spain

Claire Rogel-Gaillard

France

Hilary Rogers

UK

John Rohde

Canada

Jai Rohila

USA

Antonis Rokas

USA

Carlos Romero

Spain

Hector Romero

Uruguay

Margie Romine

USA

Philip Rosenstiel

Germany

Gil Rosenthal

USA

Mara Rosenzvit

Argentina

John Rossi

USA

Omar Rota-Stabelli

Italy

Marc Rothenberg

USA

Lisa Rowland

USA

Francesca Ruberti

Italy

Paul Rushton

USA

Steven Russell

UK

Beth Russell

USA

Hege G. Russnes

Norway

Claudia Russo

Brazil

Colm Ryan

Ireland

Ivan Rychlik

Czech Republic

Alexei Ryskov

Russian Federation

Lucia Sacchetti

Italy

Giuseppe Saccone

Italy

Gilberto Sachetto-Martins

Brazil

Timothy Sackton

USA

Jeroen Saeij

USA

David Saffen

China

Daniela Saggioro

Italy

Ozgur Sahin

USA

Harpreet Saini

UK

Ayako Sakamoto

Japan

Takashi Sakamoto

Japan

Emili Salo

Spain

Paulo Salomon

Brazil

Walter Salzburger

Switzerland

Keyan Salzman

USA

Michael Sammeth

Spain

William Sanders

USA

Ajay Sandhu

USA

Thomas Sandmann

USA

Eidy Santos

Brazil

Mauro Santos

Spain

Pablo Santos

Germany

Daniel Sargent

Italy

Mehdi Sargolzaei

Canada

Debabrata Sarkar

India

Maureen Sartor

USA

Masaki Sasai

Japan

Yasunori Sasakura

Japan

Samuel Sathyanesan Newton

USA

Kaoru Sato

Japan

Kazuhiro Sato

Japan

Yukuto Sato

Japan

Akira Satoh

Japan

Shigeru Satoh

Japan

Sampurna Sattar

USA

Barry James Saville

Canada

Samir Sawant

India

Sunita Saxena

India

Joseph Schacherer

France

Katrin Schaefer

Germany

Christoph Schaniel

USA

Manfred Schartl

Germany

Michael Schatz

USA

Mark Schembri

Australia

Laurent Schibler

France

Bernhard Schink

Germany

Jan Schirawski

Germany

Andreas Schlicker

Netherlands

Thomas Schmutzer

Germany

Gerald M. Schneeweiss

Austria

Matthias Schnell

USA

Max Schobert

Germany

Julie Scholes

UK

Uwe Scholz

Germany

Dustin Schones

USA

Jacqueline Schoumans

Sweden

Augusto Schrank

Brazil

Andreas Schreiber

Australia

Marcel H. Schulz

USA

Gadi Schuster

Israel

Lillie Searles

USA

James Seeb

USA

Lisa Seeb

USA

Bo Segerman

Sweden

Verena Seidl-Seiboth

Austria

Hank Seifert

USA

Rajandeep Sekhon

USA

Jeremy Selengut

USA

Arnab Sen

India

Francois Seneca

USA

Anirvan Sengupta

USA

Carlos Serezani

USA

Eyal Seroussi

Israel

Nicole Sessler

Canada

Praveen Sethupathy

USA

Robert Settlage

USA

Joao Setubal

Brazil

Andrew Severin

USA

Patricia Severino

Brazil

David Severson

USA

Hans-Martin Seyfert

Germany

William Shafer

USA

Zaigham Shahzad

France

Tamim Shaikh

USA

Xueyan Shan

USA

Ravi Shankar

India

Jian-Zhong Shao

China

Renfu Shao

Australia

Ning-Yi Shao

USA

Ehud Shapiro

Israel

Igor Sharakhov

USA

Soroush Sharbati

Germany

Shayan Sharif

Canada

Amit Sharma

India

Mridula Sharma

Singapore

Ram Kumar Sharma

India

Tilak Sharma

India

Thomas Sharpton

USA

Chaowen She

China

Fafu Shen

China

Ying Shen

Norway

Yiping Shen

USA

Yufeng Shen

USA

Merv Shepherd

Australia

Sue Sherman-Broyles

USA

Yuri Shestopaloff

Canada

Mingzhu Shi

USA

Qinghua Shi

China

Yun-Bo Shi

USA

Zhengli Shi

China

Tetsuo Shibuya

Japan

Shuji Shigenobu

Japan

Masayoshi Shigyo

Japan

Tokurou Shimizu

Japan

Kazuyuki Shimizu

Japan

Chuya Shinzato

Japan

Gautam Shirsekar

USA

Shin-Han Shiu

USA

Jeffry Shultz

USA

Hannah Siddle

UK

Stefan Siebert

USA

Paul Siegel

USA

Trevor Siggers

USA

Eric Sijbrands

Netherlands

Amaro Silva

Brazil

Herman Silva

Chile

Cynthia Silveira

Brazil

Francesco Silvestrini

Italy

Henner Simianer

Germany

Richard Simon

USA

Jared Simpson

UK

Ambuj Singh

USA

Rama Singh

Canada

Upinder Singh

USA

Jose Siqueira

Brazil

Marc-André Sirard

Canada

Nini Hedberg Sissener

Norway

Mikael Skurnik

Finland

Jerod Skyberg

USA

Barry Sleckman

USA

Janet Slovin

USA

Pavel Sluka

Australia

Christopher Mark Smales

UK

Craig Smith

Australia

Gilbert Smith

USA

Harold Smith

USA

Stephen Smith

USA

William Smith

USA

Michael Smoot

USA

Petr Smykal

Czech Republic

Warren Snelling

USA

Rod Snowdon

Germany

Ana Soares

Portugal

Pamela Sokol

Canada

Nick Sokol

USA

Bernd Sokolowski

USA

Leah Solberg Woods

USA

Aleksey Soldatov

Germany

Jiuzhou Song

USA

Min Ok Song

Korea, South

Xiang Song

USA

Tad Sonstegard

USA

Linda Sperling

France

Sriganesh Srihari

Singapore

Rohith Srivas

USA

Justin St. John

Australia

Gary Stacey

USA

Catherine Stamoulis

USA

Alfons Stams

Netherlands

Dana Stanley

Australia

Ian Stansfield

UK

Maud Starmans

Canada

Michael Stear

UK

Robert Steele

USA

Marja Steenman

France

John Stegeman

USA

Juan Steibel

USA

Katrina Steiling

USA

Peter Steinbacher

Austria

Kris Steinbrecher

USA

Alessandra Stella

Italy

Ulrich Stelzl

Germany

Lieven Sterck

Belgium

Burkhard Steuernagel

Germany

Susan Steyert

USA

David Still

USA

John Stingl

UK

Paul Stothard

Canada

Michael Strand

USA

Tobias Straub

Germany

Korbinian Strimmer

Germany

Philip Stuart

USA

Lisa Stubbs

USA

Bruno Studer

Denmark

David Studholme

UK

Jörg Stülke

Germany

Nancy Sturm

USA

Bing Su

China

Guosheng Su

Denmark

Zhen Su

China

Senthil Subramanian

USA

Munetaka Sugiyama

Japan

Mulpuri Sujatha

India

Paul Sumby

USA

Kim Summers

UK

Genlou Sun

Canada

Hao Sun

Hong Kong

Jingchun Sun

USA

Youting Sun

USA

Ben Sutherland

Canada

Yutaka Suzuki

Japan

Per-Arne Svensson

Sweden

Petr Svoboda

Czech Republic

Marek Switonski

Poland

W. Edward Swords

USA

Eva Sýkorová

Czech Republic

Ann-Christine Syvanen

Sweden

Michael Syvanen

USA

Aniko Szabo

USA

Sara Szabo

USA

Steven Szczepanek

USA

Gyorgy Szittya

Hungary

Joseph Szustakowski

USA

Kobi Tadmor

Israel

John Taggart

UK

Roberto Tagliaferri

Italy

Kazuhiro Takemoto

Japan

Adam Takos

Denmark

Firas Talas

Switzerland

Deborah Talkington

USA

Yutaka Tamaru

Japan

Tsuyoshi Tanaka

Japan

Chaorong Tang

China

Guiliang Tang

USA

Haixu Tang

USA

Jifeng Tang

Netherlands

Sithichoke Tangphatsornruang

Thailand

Rick Tarleton

USA

Patrick Tarpey

UK

Deirdre Ellen Kathleen Tarr

USA

Andreas Tauch

Germany

Stefan Taudien

Germany

Jennifer Taylor

Australia

Marinus Te Pas

Netherlands

Scott Tebbutt

Canada

Karsten Tedin

Germany

Marta Teixeira

Brazil

Ross Tellam

Australia

Thomas Templeton

USA

Torstein Tengs

Norway

Teija Tenhola-Roininen

Finland

Paula Tennant

Jamaica

Javier Terol

Spain

Herve Tettelin

USA

Rudolf Kurt Thauer

Germany

Charles Theillet

France

Sascha Thewes

Germany

Jyothi Thimmapuram

USA

James Thomas

USA

Kelley Thomas

USA

Russell Thomas

USA

Anne Thompson

USA

Cheryl Thompson

USA

Christopher Thompson

Germany

Claudia Elizabeth Thompson

Brazil

Cristiane Thompson

Brazil

Kai Thormann

Germany

David Threadgill

USA

Bin Tian

USA

Miaoying Tian

USA

Shiping Tian

China

Xiangjun Tian

USA

X Cindy Tian

USA

Nick Tinker

Canada

Richard Titball

UK

Basant Tiwary

India

Michèle Tixier-Boichard

France

Helena Tlaskalova-Hogenova

Czech Republic

Christian Tobias

USA

Stefan Toegel

Austria

Catherine Laure Tomasetto

France

Stan Tomavo

France

Craig Tomlinson

USA

Anni Tonteri

Finland

Bente Torstensen

Norway

Amy Toth

USA

Andrey Tovchigrechko

USA

Christopher Town

USA

Jeffrey Townsend

USA

Teruaki Tozaki

Japan

Timothy Tranbarger

France

Walther Traut

Germany

Michelle Trautwein

USA

Martin Trick

UK

Sucheta Tripathy

USA

Karina Trono

Argentina

John Trowsdale

UK

Heather True

USA

Penelope Truman

New Zealand

Kunihiro Tsuchida

Japan

Kenichi Tsuda

Germany

Kentaro Tsukamoto

Japan

Makoto Tsuneoka

Japan

Seiya Tsushima

Japan

Christopher Tuggle

USA

Mick Tuite

UK

Emily Turner

USA

Stephen John Turner

Australia

Akhilesh Tyagi

India

Benjamin Tycko

USA

Milton Typas

Greece

Argyris Tzouvelekis

Greece

Ikuo Uchiyama

Japan

Joshua Udall

USA

Yoshinobu Uemoto

Japan

Hirohide Uenishi

Japan

Saneyoshi Ueno

Japan

Cirano José Ulhoa

Brazil

Reinhard Ullmann

Germany

Elisabetta Ullu

USA

Gudrun Ulrich-Merzenich

Germany

Per Unneberg

Sweden

Graham Upton

UK

David Ussery

Denmark

Akhil Vaidya

USA

Daniel Vaiman

France

Saba Valadkhan

USA

Nicole Valenzuela

USA

Eric Vallender

USA

Miguel Valvano

Canada

Ariena Van Bruggen

USA

Bart Van De Sluis

Netherlands

Guido Van Den Ackerveken

Netherlands

Joost Van Den Brink

Netherlands

Esther Van Der Knaap

USA

Allen Van Deynze

USA

Gary Van Domselaar

Canada

Joost Van Dongen

Germany

Monique Van Hoek

USA

Hein Van Lith

Netherlands

Monique Van Oers

Netherlands

Jan Van Oeveren

Netherlands

Ildiko Van Rhijn

Netherlands

Marie-Anne Van Sluys

Brazil

Peter Van Ulsen

Netherlands

Gilles Van Wezel

Netherlands

Tom Van Wezel

Netherlands

Peter Vandamme

Belgium

Ilse Vandecandelaere

Belgium

Mario Vaneechoutte

Belgium

Paul Vanraden

USA

I Varona

Italy

Serena Varotto

Italy

Rajeev Varshney

India

Ana Tereza Vasconcelos

Brazil

Marta Vasconcelos

Portugal

Nikita Vassetzky

Russian Federation

Herve Vaucheret

France

Jose Vazquez-Boland

UK

Paola Venier

Italy

Marco Ventura

Italy

A Veronese

Italy

Pasqua Veronico

Italy

Cristina Vettori

Italy

Marcelo Vicari

Brazil

Kasey Vickers

USA

Sara Vieira-Silva

Belgium

Yves Vigouroux

France

Mathilakath Vijayan

Canada

Carlos Vilches

Spain

Martin Vingron

Germany

Charles Vinson

USA

Jaap Visser

Netherlands

Miguel Viveiros

Portugal

P Vize

Canada

Lila Vodkin

USA

Claudia Voelckel

New Zealand

Alfried Vogler

UK

Richard Vogt

USA

Christian Voigt

Germany

Silvia Von Der Heyde

Germany

John Vontas

Greece

Igor Vorechovsky

UK

Marnik Vuylsteke

Belgium

Agnieszka Waclawik

Poland

Simon Waddell

UK

Andrea Waeschenbach

UK

Virginia Walbot

USA

Aisha Walker

USA

Xiaochun Wan

China

Bing-Bing Wang

USA

Chenghui Wang

China

Haifeng Wang

China

Jianrong Wang

USA

Junwen Wang

China

Lian-Sheng Wang

China

Lipo Wang

Singapore

Mei-Hui Wang

USA

Mingyi Wang

USA

Shaolin Wang

USA

Wei Wang

USA

Xiangfeng Wang

USA

Xiao-Wei Wang

China

Xiaofei Wang

USA

Xiaojie Wang

China

XiaowuWang

China

Xiu-Jie Wang

China

Yin-Zheng Wang

China

Ying Wang

USA

Yue Wang

USA

Zhiquan Wang

Canada

Dierk Wanke

Germany

Erich E Wanker

Germany

Kevin Wanner

USA

Ilan Wapinski

USA

Honorine Ward

USA

Judson Ward

USA

Hans-Jörg Warnatz

Germany

James Wasmuth

Canada

Trudy Wassenaar

Germany

Shugo Watabe

Japan

Jakob Waterborg

USA

Grant Waterer

Australia

Robert Waterhouse

Switzerland

Jill Wegrzyn

USA

Yuanyuan Wei

China

Detlef Weigel

Germany

Eberhard Weihe

Germany

Joachim Weischenfeldt

Germany

Aalim Weljie

USA

Jie Wen

China

Lihong Weng

USA

Chris West

UK

James Whelan

Australia

Vance Whitaker

USA

Matthew White

USA

Peter White

Australia

Stefan White

Australia

Thomas Wicker

Switzerland

Saumya Wickramasinghe

USA

Ralph Wiedmann

USA

John Wiens

USA

Paul Wigley

UK

Kirk Wilhelmsen

USA

Paul Wilkinson

UK

Anne Willems

Belgium

Tim Williams

UK

Laurens Wilming

UK

Richard Wilson

USA

Klaus Wimmers

Germany

Mark Winfield

UK

Thierry Wirth

France

Keith Woeste

USA

Yuri Wolf

USA

Steven Wolinsky

USA

Emily Wong

Australia

Ben Wood

Canada

Marion Wood

New Zealand

Han Wosten

Netherlands

Agnieszka Wozniak

Belgium

Naomi Wray

Australia

Anthony Wright

Sweden

Wasco Wruck

Germany

Hao Wu

USA

Pei-FuWu

China

Shu-Biao Wu

Australia

Xiaolei Wu

USA

Yanqi Wu

USA

Yannick Wurm

Switzerland

Tobias Würschum

Germany

Anton Wutz

Austria

James Wynn

USA

James Wynne

Australia

Zhiyong Xi

USA

Guangmin Xia

China

Fengning Xiang

China

Wenzhong Xiao

USA

Chuanxiao Xie

China

Gangcai Xie

China

Zhiyong Xiong

USA

Lin Xu

USA

Chenghai Xue

China

Haiming Xu

China

Hongyan Xu

USA

Ping Xu

China

Qiang Xu

China

Xiaolei Xu

USA

Yan Xu

USA

Ying Xu

USA

Yu Xue

China

Ramon Xulvi-Brunet

USA

Gitanjali Yadav

India

Igor Yakovlev

Norway

Binnaz Yalcin

Switzerland

Toshiya Yamamoto

Japan

Yoshio Yamaoka

Japan

Jianbing Yan

Mexico

Itai Yanai

USA

Da Yang

USA

Huaan Yang

Australia

Guojun Yang

Canada

Jean Yang

Australia

Lixing Yang

USA

Rendong Yang

USA

Shuqun Yang

USA

Tae-Jin Yang

Korea, South

Xiao Yang

USA

Xiaochuan Yang

USA

Yuejian Yang

China

Zhenyun Yang

USA

Zujun Yang

China

Chen-Hsiang Yeang

Taiwan

Han Yingpeng

China

Bauke Ylstra

Netherlands

Sung Ho Yoon

Korea, South

Hiderou Yoshida

Japan

Karla Yotoko

Brazil

Douglas Young

UK

Barbara Yoza

USA

Jianming Yu

USA

Jun Yu

China

Sibin Yu

China

Tianwei Yu

USA

Xiaochun Yu

USA

Xuejie Yu

USA

Joshua Yuan

USA

Feng Yue

USA

Gen Hua Yue

Singapore

Gael Yvert

France

Mostafa Zamanian

USA

Maria Mercedes Zambrano

Colombia

Dani Zamir

Israel

Bing Zang

USA

Rafael Zardoya

Spain

Dmitri Zaykin

USA

Susanne Zeilinger

Austria

Noam Zelcer

Netherlands

Alex Zelikovsky

USA

Chang-Jun Zeng

China

Sara Zenoni

Italy

Daniel Zerbino

USA

Xiangjiang Zhan

UK

Changqing Zhang

USA

Chuan-Xi Zhang

China

Clarence K. Zhang

USA

Dapeng Zhang

USA

Hm Zhang

China

Huanmin Zhang

USA

Jinfa Zhang

USA

Julia Zhang

USA

Kui Zhang

USA

Li Zhang

USA

Meiping Zhang

USA

Shang-Hong Zhang

China

Sheng Zhang

USA

Si-Ming Zhang

USA

Tianzhen Zhang

China

Wei Zhang

USA

Weiwen Zhang

China

Xiaoyu Zhang

USA

Xiurong Zhang

China

Yong Zhang

China

Zhuohua Zhang

USA

Patrick Xuechun Zhao

USA

Qi Zhao

USA

Shu-Hong Zhao

China

Feng-Qi Zhao

USA

Wu Zhen-Fang

China

Jie Zheng

Singapore

Yizhi Zheng

China

Boxiong Zhong

China

Min Zhong

USA

Shaobin Zhong

USA

Huaijun Zhou

USA

Wengang Zhou

USA

Xiaobo Zhou

USA

Dahai Zhu

China

Guan Zhu

USA

Kun Yan Zhu

USA

Lan Zhu

USA

Mingfu Zhu

USA

Qian-Hao Zhu

Australia

Renying Zhuo

China

Agnieszka Zmienko

Poland

Jun Zou

UK

Andrea Zuccolo

Italy

